# First-ever Abscopal Effect after Palliative Radiotherapy and Immuno-gene Therapy for Malignant Pleural Mesothelioma

**DOI:** 10.7759/cureus.4102

**Published:** 2019-02-20

**Authors:** Andrew R Barsky, Keith A Cengel, Sharyn I Katz, Daniel H Sterman, Charles B Simone

**Affiliations:** 1 Radiation Oncology, Perelman School of Medicine at the University of Pennsylvania, Philadelphia, USA; 2 Radiology, Perelman School of Medicine at the University of Pennsylvania, Philadelphia, USA; 3 Internal Medicine, New York University School of Medicine, New York, USA; 4 Radiation Oncology, University of Maryland School of Medicine, Baltimore, USA

**Keywords:** mesothelioma, radiation therapy, abscopal effect, immunotherapy, gene therapy

## Abstract

Malignant pleural mesothelioma (MPM) is a highly aggressive disease, with few, if any, curative interventions. While there is growing interest in using immunotherapy and immuno-gene therapy to treat MPM, very limited data currently exist for combining these modalities with radiotherapy. Preclinical data suggest that radiotherapy may be combined with immunotherapy to produce disease regression, with abscopal effects in mice with MPM. We report the first-ever case of abscopal effect in a patient with MPM, following radiotherapy and immuno-gene therapy. The patient was a 67-year-old male with prior asbestos exposure who presented with progressive dyspnea and thoracic pain. He underwent partial right pleurectomy, pleural biopsy, and talc pleurodesis, with pathology revealing epithelioid MPM. A subsequent chest computed tomography (CT) scan and fluoro-deoxyglucose positron-emission tomography (FDG-PET) CT scan showed extensive, right-sided, fluoro-deoxyglucose (FDG) avid mass-like pleural thickening encasing the right lung, with likely mediastinal extension, nodal metastases, and vascular compression. He enrolled in a clinical trial in which he received intrapleural interferon-alpha gene therapy but needed to discontinue therapy due to supraventricular tachycardia and superior vena cava syndrome induced from tumor burden. He was emergently treated with palliative radiotherapy to 30 Gy in 10 fractions. He was then started on pemetrexed and cisplatin chemotherapy. His subsequent chest CT scan two months after radiotherapy completion showed a dramatic treatment response within, as well as outside of, the irradiated field. After completion of radiotherapy, he did experience radiation esophagitis requiring nasogastric tube placement. Herein, we highlight the feasibility and efficacy of combining immuno-gene therapy with palliative radiotherapy to produce a substantial treatment response and an abscopal effect in a patient with unresectable MPM.

## Introduction

Malignant pleural mesothelioma (MPM) is a rare, aggressive malignancy of the mesothelial surface of the pleura, with an incidence of one case per 100,000 people per year in the United States [1]. MPM has a strong causal relationship with asbestos exposure, and to a lesser extent, prior in-field radiotherapy [2] and the germline breast cancer type 1 susceptibility protein-associated protein-1 mutation [3]. The majority of MPM cases are of the epitheliod histologic subtype, which portends a more favorable prognosis [4], with the remainder composed predominantly of sarcomatoid and biphasic subtypes [5].

The management of MPM depends largely upon the patient’s staging, tumor histology, co-morbidities, and performance status. The standard of care for patients with stage IV disease and for non-epithelioid histologies is chemotherapy alone or observation for progression. The standard of care for physically fit patients with stage I-III disease and epithelioid histology is surgical resection, with or without induction or adjuvant therapies [6]. In patients with unresectable disease, definitive chemotherapy is the standard of care, with pemetrexed and cisplatin or carboplatin, with or without bevacizumab every three weeks for up to six cycles [7]. Unfortunately, even with surgery, MPM remains largely an incurable malignancy. In the definitive chemotherapy setting, a recent phase III randomized trial comparing pemetrexed, cisplatin, and bevacizumab to pemetrexed and cisplatin alone, showed a median overall survival of 18.8 months and 16.1 months, respectively, demonstrating a need for further improvement in therapeutic options [8].

 As such, there has been an interest in immunotherapy and immuno-gene therapy to treat MPM. Studies have investigated the use of interleukin-2 [9], tremelimumab [10], pembrolizumab [11], nivolumab [12], and avelumab [13] in MPM. A study of immuno-gene therapy in MPM investigated the use of a modified adenoviral vector harboring the human interferon-alpha-2b gene (Ad*.IFN*) via intrapleural infusion, followed by systemic chemotherapy, with an impressive median overall survival of 21.5 months for MPM patients with epithelial histology in the second-line setting [14]. While there is evidence that radiotherapy may independently have an immunomodulatory effect [15], and that local radiotherapy has been observed in some cases to achieve an “abscopal” effect, or radiation-induced cell death within the irradiated field, with disease regression outside of the irradiated field, potentially due to systemic immune stimulation from local radiotherapy effects [16], there currently are very few published reports combining radiotherapy with immunotherapy in MPM. Furthermore, no abscopal response has been reported to date when combining radiotherapy with immunotherapy for MPM. Herein, we report the first case of an abscopal effect for a patient with MPM treated with Ad*.IFN* immuno-gene therapy followed by radiotherapy with an exaggerated local response as well as out-of-field regression.

## Case presentation

History and physical

A 67-year-old male former smoker with a history of prior occupational asbestos exposure and recurrent bronchitis presented with progressive dyspnea and thoracic pain to the point that he could not lie down in bed. A computed tomography (CT) scan of the chest was performed, which was interpreted as right-sided pneumonia with right parapneumonic effusion. He was sent to his local emergency department, where he was admitted for antibiotics and thoracentesis, the latter which demonstrated the presence of atypical mesothelial cells with inflammatory cells. He was readmitted two weeks later for progressive thoracic pain, was found to have a recurrent right-sided pleural effusion, and was managed with partial right pleurectomy with pleural biopsy, and talc pleurodesis. Right pleural pathology demonstrated atypical mesothelial proliferation at the pleural surface, without true invasion or definitive pathologic evidence of malignancy. Following surgery, he felt substantially better, such that he could sleep in the bed again, and he was able to return to his baseline activity levels. He underwent repeat chest CT five months later, which showed right pleural thickening and a small loculated pleural effusion, favored to represent a combination of calcification, pleurodesis, and atelectasis. He remained clinically well for another five months until he presented with cough and sinus congestion unrelieved by guaifenesin, dextromethorphan, and antibiotics. He underwent repeat chest CT that showed extensive mass-like pleural thickening completely encasing the right lung, with prominent involvement of the mediastinal pleura, and probable mediastinal extension into the right paratracheal and precarinal space, with pericardial effusion and probable pericardial metastases. There was no definite invasion into the right chest wall and no evidence of disease outside of the thorax.

He then established care at our institution’s mesothelioma and pleural disease multi-disciplinary program. Pathology review of the previously biopsied pleural tumor revealed that the pleural tumor cells were positive for Wilms' tumor-1 and calretinin, and negative for mouse monoclonal epithelial cell adhesion molecule antibody and thyroid transcription factor-1, consistent with malignant epithelioid mesothelioma, invading fibro-adipose tissue. At the time of consultation, he reported increasing shortness of breath, dyspnea on exertion, intermittent cough productive of clear sputum, 40 pounds of weight loss, drenching sweats, and chest wall numbness near his incision site, although he was still able to perform rigorous exercise on a daily basis. His past medical and surgical histories were otherwise only remarkable for hypertension and inguinal hernia repair surgery, respectively. His family history was notable for mesothelioma in a maternal uncle and breast cancer in a maternal aunt. His exam revealed diffusely decreased right-sided breath sounds, right-sided dullness to percussion, a well-healed right chest wall incision, and an Eastern Cooperative Oncology Group (ECOG) performance status of one. His forced vital capacity (FVC) was 1.88 L (41% of predicted) and forced expiratory volume in one second (FEV1) was 1.54 L (46% of predicted).

**Figure 1:**
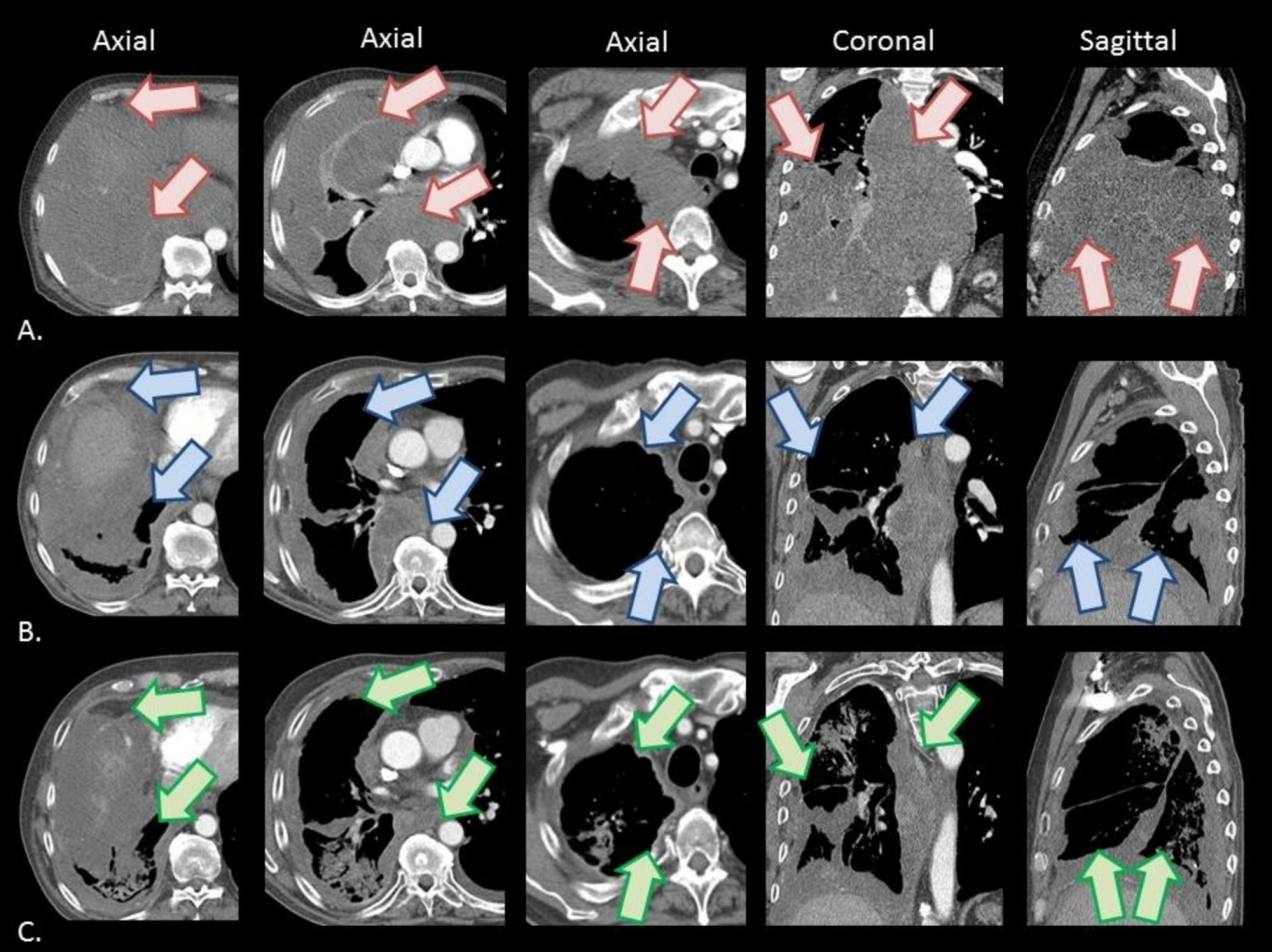
Pre and post-treatment chest CT scans Axial (from left to right, at the levels of the diaphragm, great vessels, and inferior clavicular heads), coronal, and sagittal IV contrast-enhanced CT images obtained (A) prior to immuno-gene therapy, and at (B) three months and (C) six months following immuno-gene therapy, revealing a sustained dramatic decrease in burden of pleural tumor. Representative regions of the tumor prior to immuno-gene therapy are indicated by red arrows, with representative regions of tumor response to therapy at three months and six months following immuno-gene therapy indicated by blue arrows and green arrows, respectively. CT, computed tomography; IV, intravenous

He was seen by pulmonology, medical oncology, radiation oncology, and thoracic surgery, with further staging recommended. Fluoro-deoxyglucose positron-emission tomography (FDG-PET) CT was performed and showed extensive mass-like circumferential pleural thickening throughout the right hemithorax which was diffusely fluoro-deoxyglucose (FDG) avid (SUVmax 16.1) and invaded the left hilum with mass effect on the left atrium. It also showed diffuse metastatic nodularity of the pericardium and associated small pericardial effusion. Magnetic resonance imaging (MRI) of the brain was negative for metastatic disease. A repeat chest CT with intravenous contrast re-demonstrating the known disease burden but was concerning for vascular invasion (Figure 1A). An MRI of the chest showed a 19.4 x 20.8 x 21.5-cm mass in the right pleural space with mediastinal and diaphragmatic invasion. The tumor extended across the midline at the level of the carina, with vascular encasement, segmental esophageal encasement, the involvement of the azygoesophageal recess, and invasion into the superficial right hemidiaphragm. As a result of his clinical American Joint Committee on Cancer primary tumor stage T4 disease due to the mediastinal invasion, he was not a surgical candidate. He opted to enroll in phase I/II clinical trial of front-line pemetrexed/cisplatin chemotherapy in combination with repeated dose intrapleural interferon-alpha gene therapy [14].

Intrapleural interferon-alpha immuno-gene therapy details

The patient went to the operating room for surgical creation of a small pleural space in which a pleural catheter was placed, approximately 5 cm below the tip of the right scapula in the lateral chest wall, to facilitate intrapleural infusion of the adenoviral vector for immuno-gene therapy, as per study protocol. The following week, he received his first intrapleural interferon-alpha gene therapy treatment, which he tolerated without complication. Five days later, he presented for his second treatment, at which time he developed sudden tachycardia and electrocardiogram revealed supraventricular tachycardia (SVT) requiring treatment with adenosine. As a result, his second intrapleural treatment was held. The following day, he experienced another episode of SVT with hypotension, for which he again responded to adenosine. He was admitted to the hospital, where a chest CT revealed significant compression of the pulmonary veins and left atrium and transthoracic echocardiogram demonstrated increasing pulmonary hypertension, with mild right ventricular dilation. Cardiology was consulted, who attributed the cardiac symptoms to vascular compression. The options of urgent chemotherapy or radiotherapy were discussed, and radiotherapy was recommended in an attempt to quickly improve symptoms and relieve vascular compression.

Radiotherapy setup and treatment planning details

**Figure 2:**
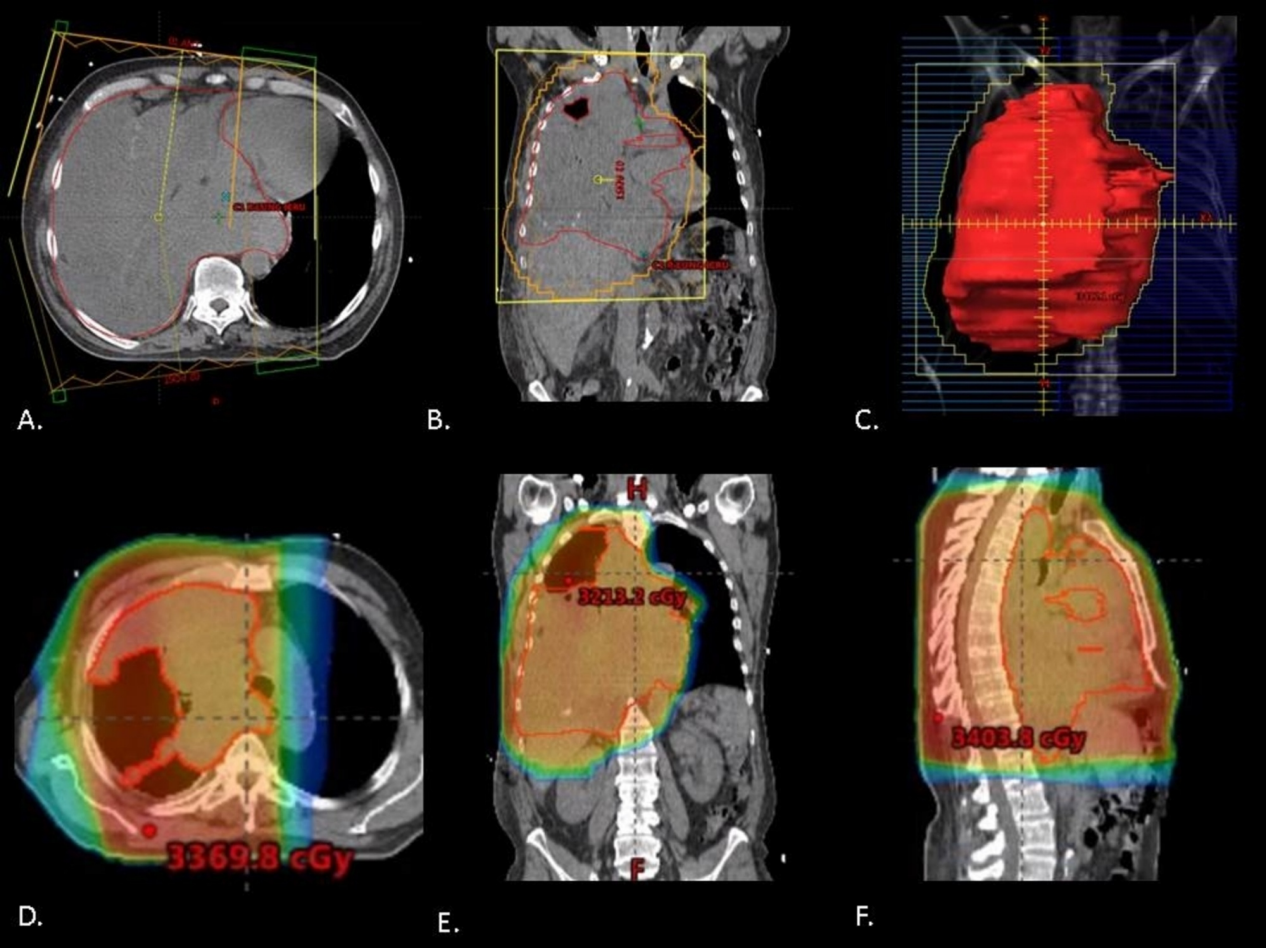
Radiation treatment plan Representative axial (A) and coronal (B) simulation CT images depicting the radiotherapy fields used for treatment, with the GTV outlined in red. Digitally reconstructed radiograph of an anterior-posterior field with multi-leaf collimators used for treatment, with the GTV outlined in red (C). Representative axial (D), coronal (E), and sagittal (F) simulation CT images depicting radiotherapy dose distribution for the treated plan. A dose of 30 Gy was delivered in 10 fractions using forward-planned, field-in-field three-dimensional conformal radiotherapy. CT, computed tomography; GTV, gross tumor volume

The patient underwent CT simulation in the supine position with his arms above his head, with a full body vac-lock bag used for immobilization. The isocenter was set at the carina. The primary tumor was contoured using the simulation CT dataset, with the gross tumor volume (GTV) defined as all visible tumor on the simulation CT images. A 0.8-mm expansion to account for the microscopic disease was added to the GTV to generate the clinical target volume (CTV). The planning target volume (PTV) was generated as a 0.5-cm uniform expansion on the GTV. The plan was designed to treat the PTV to a dose of 30 Gy in 10 daily fractions using a forward-planned, field-in-field, three-dimensional conformal radiotherapy (3D-CRT) technique. A two-field technique was used with 15-MV anterior-posterior and posterior-anterior beams and multi-leaf collimation (MLC) to spare dose to bone, heart, liver, and kidney. Image-guided radiotherapy, with daily pre-treatment kV-kV scans, was used to optimize patient alignment and target localization. The treatment volumes and plan are depicted in Figure 2.

Radiotherapy course

His treatment was tolerated well overall, without any unplanned breaks. His pre-radiotherapy dyspnea improved rapidly during treatment, such that he no longer required supplemental oxygen by the second week of treatment. He developed acute grade two dyspepsia, managed with omeprazole, as well as grade two dysphagia, cough, fatigue, and constipation. His ECOG performance status improved from three at the start of radiotherapy to two at the end of radiotherapy.

Post-radiotherapy course

Two days after completion of radiotherapy, he was started on systemic therapy with intravenous pemetrexed (500 mg/m^2^) and cisplatin (75 mg/m^2^). Six days after cycle one day one of chemotherapy, he noted severe radiation esophagitis and weight loss, prompting hospitalization, during which time he required nasogastric tube (NGT) feeds and opioid analgesia. His symptoms improved over that week, and his NGT was removed. He received cycles two and three of chemotherapy, 21 days apart, with post-cycle three chest CT showing dramatic interval response to therapy, with a representative mass in the right paratracheal region measuring 1.2 x 8.5 cm, previously 3.9 x 9.1 cm two months prior (Figure 1B). He continued with another three cycles of chemotherapy, with repeat chest CT after cycle six showing ongoing interval improvement of multiple pleural masses, with profound response in the ipsilateral treated hemi-thorax, and no evidence of contralateral pericardial disease. Overall, he tolerated these six cycles of systemic therapy well and reported improvements in energy level, appetite, breathing, and odynophagia.

The patient then opted to enroll on a randomized trial of maintenance pemetrexed versus observation following completion of six cycles of first-line therapy [NCT01085630], and he was randomized to the pemetrexed arm. He continued on pemetrexed monotherapy (500 mg/m^2^) for a total of eight cycles, every 21 days, over the next seven months. Following maintenance cycle two, his chest CT showed ongoing stable disease (Figure 1C). He tolerated this well initially, with occasional fatigue, anemia, ankle edema, and cough. During cycle four, he reported some productive cough and worsening dyspnea on exertion with subsequent chest CT scan revealing increasing multifocal opacities involving nearly all lobes of the lung. These imaging findings were favored to represent infection versus delayed radiation pneumonitis versus chemotherapy-induced pneumonitis, for which he was managed with levofloxacin and prednisone, with significant improvement of symptoms. He initially clinically improved on this therapy, however ultimately required admission to the intensive care unit following cycle six for septic shock attributed to right-sided pneumonia, which improved with volume resuscitation, antibiotics, and steroids. His CT chest at the time of admission demonstrated a stable burden of mesothelioma. After discharge home, the patient continued with cycles seven and eight of pemetrexed but was admitted about one month after his final cycle for worsening lower extremity edema, dyspnea, and orthopnea that was only partially responsive to intravenous diuretics. His chest CT at this time showed increasing consolidation in the right lung, with stable pleural thickening, but with an increasing left-sided pleural effusion without pleural thickening. Given a negative workup by cardiology, and a lack of other clear explanations for his symptoms and contralateral effusion, this clinical worsening of symptoms was favored to represent disease progression, and he was switched to treatment with gemcitabine (1000 mg/m^2^), of which he received two cycles of therapy.

Unfortunately, in the first few weeks following gemcitabine therapy, he developed progressive shortness of breath and performance status decline. Reimaging confirmed that he had further progressive disease. Systemic therapy was stopped, and he was transitioned to outpatient hospice. He passed away comfortably three months later.

## Discussion

To date, this is the first published report of an abscopal effect in a patient with MPM, as well as the first report of abscopal effect following radiotherapy and immuno-gene therapy for MPM. While this was not the initially planned treatment regimen for the patient, it is noteworthy to report the findings given the novelty of immuno-gene therapy, low radiotherapy dose, and substantial, durable local response to therapy for this aggressive disease.

In this patient’s case, the initial plan was to proceed with two infusions of immuno-gene therapy, as per the study protocol [14]. However, given that he developed SVT requiring adenosine infusion prior to the second planned infusion, the remainder of his immuno-gene therapy course was discontinued. Per the study protocol, should he have completed his two infusions, the next therapy would have been standard chemotherapy. However, he developed critical findings concerning for superior vena cava syndrome, and given his worsening illness, he was emergently started on a course of palliative radiotherapy, with the hope that this would prevent further immediate progression of disease, and potentially also regression of disease. As can be seen in Figure 1, the patient’s response to immuno-gene therapy, palliative radiotherapy, and subsequent chemotherapy, both in-field and out-of-field was dramatic, and notably more pronounced than what is generally achievable for even well-responding MPM patients. In fact, his exaggerated response was far greater than would be expected based upon the volume of disease irradiated and radiation dose employed, potentially by eliciting an abscopal effect.

The hypothesis for the mechanism of action for the abscopal effect is that tumor cell injury by radiation therapy generates the release of tumor cell neoantigens which are then taken up by antigen-presenting cells and brought to lymph nodes to activate T-cells. There, T-cells become primed to identify and attack tumor-specific antigens, allowing them to attack tumor that expresses these antigens, which includes both the treated primary and out-of-field tumor, leading to tumor regression both in-field and out-of-field. In the last few decades, there has been increasing recognition of the abscopal effect, such that 46 case reports of this type of tumor response from radiotherapy alone have been published between 1969 and 2014. The tumor types in which an abscopal response has been described include hepatocellular carcinoma, melanoma, and renal cell carcinoma, among others [17]. To our knowledge, this case represents the first reported case of an abscopal effect in MPM.

Given the dismal prognosis of MPM, even with the best available standard modern therapies, there is interest in using novel therapies, such as immunotherapy, or immuno-gene therapy, in its treatment. Given the efficacy of therapies directed against cytotoxic T-lymphocyte-associated protein 4 (CLTA-4) in other cancers, tremelimumab, an anti-CTLA-4 antibody, was studied in a randomized phase III trial against placebo in MPM, but it unfortunately did not show a significant improvement in overall survival (7.7 vs. 7.3 months) [10]. Therapies directed against programmed cell death protein 1 (PD-1) and programmed cell death-ligand 1 (PD-L1) have also been studied in MPM [18]. Pembrolizumab, an anti-PD-1 antibody, was used in a phase I study of 25 PD-L1-positive patients with MPM and yielded an overall response rate of 20%, with more than half of the patients having stable disease for over six months [11]. Nivolumab, also an anti-PD-1 antibody, was used in a phase II study of 34 patients with MPM and showed a 50% disease control rate at 12 weeks [12]. Avelumab, an anti-PD-L1 antibody, was used in a phase I study of patients with 53 patients who received prior treatment for unresectable MPM or malignant peritoneal mesothelioma, and it was found to have nearly a 10% partial response rate, with a and median progression-free survival of 17.1 weeks, and a safe toxicity profile [13]. In addition, immuno-gene therapy for MPM with intrapleural Ad.IFN therapy, followed by systemic chemotherapy, has been studied, as in this patient, with a median overall survival of 21 months for the whole epithelial cohort [14]. While recognizing that this was not a randomized trial and should be compared to other survival data with caution, it is worth noting that an overall survival of 21.5 months among second-line patients with this novel combination compares favorably to that of standard of care definitive chemotherapy with pemetrexed, cisplatin, and bevacizumab for unresectable MPM among even first-line patients, which yielded a median overall survival of 18.8 months [8]. As these relatively new data emerge demonstrating safety and some degree of efficacy for immunotherapy and immuno-gene therapy, increasing interest is emerging in understanding how these therapies interact with radiotherapy.

The possibility exists that radiotherapy could be combined with these systemic treatments to produce an augmented radiotherapy effect, and possibly, an out-of-field abscopal effect. Preclinical data exist that suggest that radiation can act synergistically with immune checkpoint inhibitors by increasing tumor antigen production and presentation, increasing cytotoxic T-lymphocyte activity, and decreasing myeloid immune suppressor cells. It has also been suggested that radiation-associated immune activation could lead to a more thorough regression of the irradiated tumor, as well as microscopic metastatic disease that was not appreciated at the time of initial therapy [19]. In a murine study of localized radiotherapy and anti-CTLA-4 therapy in an MPM model, the growth of the primary tumor as well as the non-irradiated tumor was delayed. Further analysis of the mice showed increased Treg and cytotoxic T-cell infiltration in the primary and non-irradiated tumor, with subsequent decreases in Treg cells and increases in cytotoxic T-cells after anti-CTLA-4 therapy was administered, suggesting the feasibility of the abscopal effect in MPM [18]. In humans, the data for combining immunotherapy with radiotherapy are more limited but appear promising in thoracic malignancies like non-small cell lung cancer [20], as well as extra-thoracic malignancies [19]. That said, this is an active area of interest, with ongoing clinical trials studying the combination of immunotherapy with radiotherapy in non-small cell lung cancer, small cell lung cancer, esophageal cancer, and MPM, among others [20]. Herein, we provide evidence that immuno-gene therapy combined with radiotherapy in MPM was feasible and capable of producing durable locoregional and distant disease control, as well as abscopal effect, for this highly aggressive malignancy.

While effective, the toxicity of therapy in this patient warrants discussion. The patient received his first immuno-gene therapy infusion without issue, but within a few days developed two episodes of SVT with hypotension. Following evaluation by the cardiology team, it was favored that tumor burden with resultant vascular compression was the most likely etiology of his symptoms, but given the temporal relation to his infusion, the relationship to the immunotherapy cannot be definitively excluded. It is possible that the SVT may have been a result of an intra-tumoral inflammatory response in the setting of recent immuno-gene therapy infusion, with bulky tumor adjacent to the pericardium. As such, there may need to be additional caution in the future with Ad.IFN administration in similar settings of bulky disease adjacent to vital structures. Furthermore, while he tolerated radiotherapy without issue during treatment, he went on to develop severe radiation esophagitis with weight loss, requiring NGT placement and hospitalization. While radiation esophagitis is an expected side effect of thoracic radiotherapy, even with a relatively low dose of 30 Gy in 10 fractions, severity to the point of NGT placement from radiotherapy alone is greater than expected. As such, it is possible that having received recent immuno-gene therapy may have made him more susceptible to the development of inflammatory side effects. It is also possible that his innately being susceptible to radiation-induced inflammatory toxicities also immunologically allowed for him to respond better to immunotherapy and/or be more likely to develop an abscopal effect from the combination of radiation therapy and systemic therapy. While the development of these adverse effects should not preclude therapy in appropriately selected patients, it is worthwhile to know that these effects can happen and to counsel patients and providers to be aware of such symptoms, such that appropriate surveillance and early intervention can be administered. Further study of immunotherapy and immuno-gene therapy with radiotherapy, with differing dose and fractionation schemas, both in MPM and other disease sites is warranted.

## Conclusions

MPM is a highly aggressive disease with few, if any, curative interventions, and thus a need exists for improved therapies, including immunotherapy and immuno-gene therapy. With this case report, we provide evidence that immuno-gene therapy combined with radiotherapy in MPM is feasible and capable of producing durable locoregional and distant disease control, as well as abscopal effect.
